# Support and approximation properties of Hermite splines

**DOI:** 10.1016/j.cam.2019.112503

**Published:** 2020-04

**Authors:** Julien Fageot, Shayan Aziznejad, Michael Unser, Virginie Uhlmann

**Affiliations:** a Biomedical Imaging Group, École polytechnique fédérale de Lausanne (EPFL), Station 17, 1015 Lausanne, Switzerland; b European Bioinformatics Institute (EMBL-EBI), Wellcome Genome Campus, Cambridge CB10 1SD, UK

**Keywords:** Hermite interpolation, Minimum-support property, Approximation error

## Abstract

In this paper, we formally investigate two mathematical aspects of Hermite splines that are relevant to practical applications. We first demonstrate that Hermite splines are maximally localized, in the sense that the size of their support is minimal among pairs of functions with identical reproduction properties. Then, we precisely quantify the approximation power of Hermite splines for the reconstruction of functions and their derivatives. It is known that the Hermite and B-spline approximation schemes have the same approximation order. More precisely, their approximation error vanishes as $O\left(T^{4}\right)$ when the step size T goes to zero. In this work, we show that they actually have the same asymptotic approximation error constants, too. Therefore, they have identical asymptotic approximation properties. Hermite splines combine optimal localization and excellent approximation power, while retaining interpolation properties and closed-form expression, in contrast to existing similar functions. These findings shed a new light on the convenience of Hermite splines in the context of computer graphics and geometrical design.

## 1 Introduction

In his seminal 1973 monograph on cardinal interpolation and spline functions [1], I.J. Schoenberg explains and characterizes B-spline interpolation, which still inspires researchers and yields exciting applications nowadays. In the same work, he also sets the basis of Hermite interpolation [2], [3]. In the classical B-spline framework, a continuous-domain function is constructed from a discrete sequence of samples [4], [5], [6]. By contrast, the Hermite interpolation problem involves two sequences of discrete samples. They impose constraints not only on the resulting interpolated function but also on its derivatives up to a given order.

Curves in the plane or tensor-product surfaces in space can be constructed from one-dimensional interpolation schemes by interpolating along each spatial coordinate. The practical value of Hermite splines in this context is to offer tangential control on the interpolated curve. This can be easily understood through their link with Bézier curves [7]. The latter lie at the heart of vector graphics and are popular tools for computer-aided geometrical design and modeling [8], [9], [10]. Because of their small support, Hermite splines are also an interesting option for the design of multiwavelets, which are wavelets with multiple generators [11], [12]. In practice, Hermite splines thus provide a suitable solution to a number of problems, whether with respect to simplicity of construction, efficiency, or convenience. This hands-on intuition can be translated to formal properties of Hermite splines and mathematically characterized. Wer give as examples the joint interpolation properties of Hermite splines (see Section 1.2) that ensure that, at integer values, the interpolated function exactly matches the sequences of samples and derivative samples that were used to build it; their smoothness properties [13], which guarantee low curvature of the interpolated curve under some mild conditions; and their statistical optimality (in terms of MMSE) for the reconstruction of second-order Brownian motion from direct and first-derivative samples [14]. In that spirit, we investigate in this work the theoretical counterpart of two additional features that are observed to grant Hermite splines their practical usefulness.

### 1.1 Contributions

Our contributions state the minimal-support property of Hermite splines and investigate their approximation power. In the following, we describe the practical observations that motivate them, the results themselves, and related works.

#### Minimal-support property.

The short support of Hermite splines is an important feature that makes them attractive in practice. The size of the support relates to the local extent of modifications on the continuously defined spline curve. In Section 2, we formally demonstrate that Hermite splines have the minimal support among all basis functions that generate cubic and quadratic splines. When dealing with B-splines, there is a tradeoff between the ability to reproduce smooth functions, which increases with the B-spline polynomial degree, and the possibility to allow for more or less sharp transitions, which decreases with the degree. As the question of function reproduction is a central concept when studying both minimal support and approximation errors, we provide a formal definition of it in Section 2.1. On one hand, cubic splines can efficiently reproduce smooth functions are able to capture $C^{2}$ transitions, but lack the power to capture $C^{1}$ transitions. On the other hand, quadratic splines have a lesser approximation power, but are preferred when dealing with less smooth ($C^{1}$) transitions. Hermite splines combine these two strengths in one scheme and are, in terms of support size, better than two-function schemes, including the one composed of the classical cubic and quadratic B-splines. In addition, we also show that one necessarily requires two generators to achieve this optimality. This result relates to similar ones involving a single generator [15], [16].

#### Rate of decay of the approximation error.

Hermite splines can provide faithful approximation reasonably fast as the number of parameters increases. This feature relates to the rate of decay of the error of approximation. Numerous works approach these questions by restricting themselves to a specific interpolation framework, such as [17], [18] for the specific case of Hermite approximation. Relying on $L_{\infty }$ norms, they provide precise estimations of the optimal bound on the approximation error, but do not allow for comparisons with other schemes. In contrast, approaches have been developed for single [19] and multiple generators [20] to provide a unifying comparison setting. They offer generalized measures that can be applied to a wide variety of basis functions to estimate approximation constants. Hermite interpolation, however, violates some of the core assumptions of those analyses. There, we take strong inspirations from those previous works and provide a novel study of Hermite approximation. We deploy an analogous analysis strategy by relieving the boundedness assumptions and considering additional spline approximation schemes. In Section 3, we precisely quantify the rate of decay of the error of approximation of the Hermite scheme and quantitatively estimate the corresponding approximation constants. Hermite-spline interpolation offers excellent approximation properties when it comes to the reconstruction of a function and its first derivative. It is actually close to achieving the minimal error obtained when the approximation procedure is modified to correspond to the orthogonal projector. The investigation of the error of approximation on the derivative relates to [21], [22], although it follows a completely different line: while [21], [22] focused on the reconstruction of the derivative from signal samples, the Hermite scheme grants direct access on the function and derivative samples, allowing one to reconstruct the derivative in a multifunction setting.

Pioneering works cover the study of spline schemes to an impressive degree of generality [23], [24], [25], [26]. They include results on minimum support and approximation errors in a large variety of cases. Regarding minimum support, these previous results are however restricted to single-generator schemes. To the best of our knowledge, there are no previous works that would consider multi-generators and cover the Hermite case. These pioneering works also do not focus on providing a way to quantify the approximation-error constants, thus preventing one to compare schemes of the same order.

A better understanding of the approximation and support properties of Hermite splines has several useful consequences. For instance, wavelet schemes are commonly built from Hermite splines [27], [28], so that the precise characterization of the approximation power of wavelet bases is important in practical applications such as image compression [29]. Hermite splines are also used to construct parametric deformable contours in the context of image segmentation [13], [30], where their small support allows for the representation of open curves with natural conditions at their extremities [31].

### 1.2 Hermite splines

Schoenberg defines the cardinal cubic-Hermite-interpolation problem as follows [2], [3]: knowing the discrete sequences of numbers c[k] and d[k], k∈Z, we look for a continuously defined function $f_{\mathrm{Her}}\left(t\right)$, t∈R, that satisfies $f_{\mathrm{Her}}\left(k\right)=c\left[k\right]$, $f_{\mathrm{Her}}^{\prime }\left(k\right)=d\left[k\right]$ for all k∈Z, such that $f_{\mathrm{Her}}$ is piecewise polynomial of degree at most 3 and once differentiable with continuous derivative at the integers. The existence and uniqueness of the solution is guaranteed [2, Theorem 1] for any sequences c=(c[k]) and d=(d[k]) bounded by a polynomial, but we shall restrict to sequences in $\ell_{2}\left(Z\right)$ thereafter. In [3], it is shown that the Hermite spline $f_{\mathrm{Her}}$ associated to the sequences c and d can be expressed as

(1) $$f_{\mathrm{Her}}\left(t\right)=\underset{k\in Z}{\sum }c\left[k\right]\phi_{1}\left(t-k\right)+d\left[k\right]\phi_{2}\left(t-k\right),$$

where the functions $\phi_{1}$ and $\phi_{2}$ are given by

(2) $$\phi_{1}\left(t\right)=\left(2\left|t\right|+1\right){\left(\left|t\right|-1\right)}^{2}1_{0\leq \left|t\right|\leq 1},$$

(3) $$\phi_{2}\left(t\right)=t{\left(\left|t\right|-1\right)}^{2}1_{0\leq \left|t\right|\leq 1}.$$

**Fig. 1:**
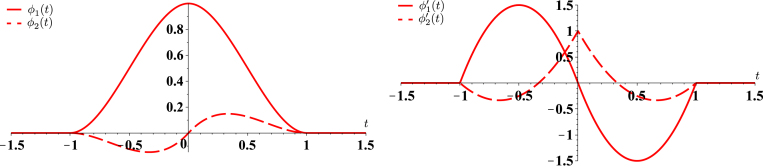
Cubic Hermite splines $\phi_{1}$ and $\phi_{2}$. The two functions and their derivatives are vanishing at the integers, with the exception of $\phi_{1}\left(0\right)=1$ and $\phi_{2}^{\prime }\left(0\right)=1$ (interpolation properties). They are supported in [−1,1].

In addition to their fairly simple analytical expression, the cubic Hermite splines have other important properties. First, they are of finite support in [−1,1]. Moreover, the generating functions $\phi_{1}$, $\phi_{2}$ and their derivatives $\phi_{1}^{\prime }$, $\phi_{2}^{\prime }$ satisfy the joint interpolation conditions

(4) $$\phi_{1}\left(k\right)=\delta \left[k\right],\;\phi_{2}^{\prime }\left(k\right)=\delta \left[k\right],\;\phi_{1}^{\prime }\left(k\right)=0,\;\phi_{2}\left(k\right)=0,$$

for all k∈Z, where δ[k] is the discrete unit impulse. The functions and their first derivative are depicted in Fig. 1, where the interpolation properties can easily be observed. The functions $\phi_{1}$ and $\phi_{2}$ are deeply intertwined as $c\left[k\right]=f_{\mathrm{Her}}\left(k\right)$ and $d\left[k\right]=f_{\mathrm{Her}}^{\prime }\left(k\right)$ in (1). The cubic Hermite splines are differentiable with continuous derivatives at the integer knots points t=k. As a result, functions generated by cubic Hermite splines are $C^{1}$-continuous piecewise-cubic polynomials with knots at integer locations.

## 2 Minimum-support property of Hermite splines

B-splines are known to be maximally localized, meaning that they are compactly supported with minimal-support property among functions with the same approximation properties [32], [33]. Hermite splines possess a similar fundamental minimal-support property: they are of minimal support among the pair of functions that generate both quadratic and cubic splines (Theorem 3). In addition, there exists no single generating function that can take this role (Proposition 5). This demonstrates that Hermite splines are maximally localized for the purpose of representing piecewise-cubic functions that are continuously differentiable, as exploited, for instance, in image processing for the design of deformable parametric contours [13].

### 2.1 Integer-shift-invariant spaces and support properties

Consider a set of N≥1 functions $\varphi =\left(\varphi_{1},\ldots ,\varphi_{N}\right)\in {\left(L_{2}\left(R\right)\right)}^{N}$ and define the space of functions

(5) $$V\left(\varphi \right)=\left\{\overset{N}{\underset{i=1}{\sum }}\underset{k\in Z}{\sum }c_{i}\left[k\right]\varphi_{i}\left(\cdot -k\right):c=\left(c_{1},\ldots ,c_{N}\right)\in {\left(\ell_{2}\left(Z\right)\right)}^{N}\right\}.$$

We say that the $\varphi_{i}$ are *basis functions* of the set V(φ). The space V(φ) is *integer-shift-invariant* in the sense that f(⋅−k) is in V(φ) for f∈V(φ) and k∈Z [23], [24], [25], [34].

We consider integer-shift-invariant spaces generated by a single (N=1) or two functions (N=2), which corresponds to B-splines with simple knots and Hermite splines, respectively. We only consider spaces (5) for which the family ${\left(\varphi_{i}\left(\cdot -k\right)\right)}_{i=1\ldots N,k\in Z}$ is a Riesz basis and satisfies

(6) $$A\overset{N}{\underset{i=1}{\sum }}\underset{k\in Z}{\sum }c_{i}{\left[k\right]}^{2}\leq {\left\|\overset{N}{\underset{i=1}{\sum }}\underset{k\in Z}{\sum }c_{i}\left[k\right]\varphi_{i}\left(\cdot -k\right)\right\|}_{L_{2}}^{2}\leq B\overset{N}{\underset{i=1}{\sum }}\underset{k\in Z}{\sum }c_{i}{\left[k\right]}^{2}$$

for any $c_{i}\in \ell_{2}\left(Z\right)$ with i=1,…,N and for some positive and finite constants 0<A≤B<∞. This ensures that any f∈V(φ) has a unique and stable representation in the basis ${\left(\varphi_{i}\left(\cdot -k\right)\right)}_{i=1\ldots N,k\in Z}$.

Any function f∈V(φ) can be *reproduced* by the family ${\left(\varphi_{i}\left(\cdot -k\right)\right)}_{i=1\ldots N,k\in Z}$ in the sense that there exist sequences $c_{i}\in \ell_{2}\left(Z\right)$ such that $f=\sum_{i,k}c_{i}\left[k\right]\varphi_{i}\left(\cdot -k\right)$. However, such functions are necessary in $L_{2}\left(R\right)$ according to (6). It is interesting to investigate the reproduction properties beyond $L_{2}\left(R\right)$; in particular, the ability to reproduce polynomials. For this purpose, one needs to extend the notion. We say that a function f can be *reproduced* by ${\left(\varphi_{i}\left(\cdot -k\right)\right)}_{i=1\ldots N,k\in Z}$ if there exists sequences $c_{i}$ such that $f\left(t\right)=\sum_{i,k}c_{i}\left[k\right]\varphi_{i}\left(t-k\right)$ holds for all t∈R. What is hidden here is that the sum should be well-defined at any time t. When the basis functions are compactly supported, the sum is actually finite for every fixed t∈R and any sequence $c_{i}$. In this section, we restrict ourselves to compactly supported basis functions since our goal is to characterize basis functions of minimal support. For non-compactly supported functions, the same notion is valid up to the condition that the sum is absolutely convergent for any t∈R. The reproduction of polynomials will play a crucial role all along the paper. It is formalized in Definition 1.

**Definition** 1 A family of N≥1 basis functions $\varphi =\left(\varphi_{1},\ldots ,\varphi_{N}\right)$ is *of order* L if they satisfy $\int_{R}{\left(1+\left|t\right|\right)}^{L}\left|\varphi_{n}\left(t\right)\right|dt<\infty$ for 1≤n≤N and if they can reproduce polynomial functions up to degree (L−1), meaning that, for ℓ=0,…,(L−1), there exist sequences $c_{i,\ell }$ such that

(7) $$\forall t\in R,\;t^{\ell }=\overset{N}{\underset{i=1}{\sum }}\underset{k\in Z}{\sum }c_{i,\ell }\left[k\right]\varphi_{i}\left(t-Nk\right).$$

It is worth noting that the sequence of coefficients is not required to take the values of the polynomial at the knots but, instead, that the interpolated function and the polynomial coincide. Condition (7) is known to be equivalent to the so-called Strang and Fix conditions and is very classical in approximation theory [35]. The hypothesis that $\int_{R}{\left(1+\left|t\right|\right)}^{L}\left|\varphi_{n}\left(t\right)\right|dt<\infty$ ensures that the Fourier transform of the $\varphi_{n}$ admits continuous and bounded derivatives up to order L, which is actually crucial in the Stang and Fix formulation. This technical condition appears and is discussed in [20, Section II-D]. Note that it is clearly satisfied as soon as the functions are compactly supported and locally integrable, as will be the case in the rest of this section. In Section 3, we shall consider basis functions that are possibly non-compactly supported.

#### Support of B-splines.

Under the Riesz condition, a natural question is the ability of the basis functions φ to exactly reproduce classes of functions. The possibility to perfectly reproduce polynomials is of crucial importance. The constant function 1 can be reproduced if and only if the basis functions satisfy the partition of unity, which is the minimal requirement for a practical approximation scheme [36].

The polynomial B-spline of order L≥1 is classically known to be able to reproduce polynomials $t^{\ell }$ for every ℓ=0,…,(L−1). Following [37], we denote it by $\beta^{\left(L-1\right)}$. In particular, the cubic B-spline (of order L=4) can perfectly reproduce any polynomial of degree at most 3. The ability of the basis functions φ to perfectly reproduce polynomials is intimately linked to their approximation power, as will be developed in Section 3. B-splines are actually the most localized functions that satisfy this property, as formalized in Proposition 2.

Proposition 2 *Let* $\varphi \in L_{2}\left(R\right)$ *be a compactly supported function such that* ${\left(\varphi \left(\cdot -k\right)\right)}_{k\in Z}$ *is a Riesz basis and can reproduce polynomials up to degree*(L−1)≥0*. Then, the support of* φ *is at least of size* L*.*

*In particular, the B-spline of order* L*, whose support is of size* L*, is optimal in terms of support localization among basis functions that are able to reproduce polynomials of degree at most* (L−1)*.*

This result is classical in approximation theory: Schoenberg showed that B-splines effectively have the adequate approximation order [1]. A complete characterization of the piecewise-polynomial functions of minimal support with a given approximation order can be found in [16, Theorem 1]. To the best of our knowledge, very little is known about the localization of basis functions when N>1, which is what we propose to investigate. In the multifunction scheme, we shall characterize reproduction properties by considering the reproduction of B-splines instead of polynomials. This takes advantage of the well-known reproduction properties of B-splines, which are inherited by any family that is able to reproduce them.

### 2.2 Minimal-support properties for two basis functions

The Hermite splines $\phi_{1}$ and $\phi_{2}$, given by (2), (3), are able to reproduce both $\beta^{2}$ and $\beta^{3}$, the quadratic and cubic B-splines of order 3 and 4, respectively [13]. In particular, this means that $V\left(\phi_{1},\phi_{2}\right)$ contains polynomials of degree at most 3. Many other pairs of basis functions, starting with $\beta^{2}$ and $\beta^{3}$ themselves, can also reproduce quadratic and cubic splines. In line with Proposition 2, the investigation the support of a pair of functions that have the same reproduction properties as the Hermite splines follows naturally. This boils down to the study of basis functions for which $\beta^{2},\beta^{3}\in V\left(\varphi_{1},\varphi_{2}\right)$.

We characterize the support of such pairs of functions in Theorem 3. This result then allows us to deduce the minimal-support property of Hermite splines in Corollary 4.

**Theorem** 3 *Let* $\varphi_{1},\varphi_{2}\in L_{2}\left(R^{d}\right)$ *be two compactly supported basis functions. We assume that*

(8) $$\beta^{2}\left(t\right)=\underset{k\in Z}{\sum }a_{k}\varphi_{1}\left(t-k\right)+b_{k}\varphi_{2}\left(t-k\right),$$

(9) $$\beta^{3}\left(t\right)=\underset{k\in Z}{\sum }c_{k}\varphi_{1}\left(t-k\right)+d_{k}\varphi_{2}\left(t-k\right),$$

*with reproduction sequences* a,b,c,d *that satisfy*

(10) $$\underset{k\in Z}{\sum }k^{3}\left(\left|a_{k}\right|+\left|b_{k}\right|+\left|c_{k}\right|+\left|d_{k}\right|\right)<\infty .$$

*In particular, the quadratic and cubic B-splines* $\beta^{2},\beta^{3}$ *are in* $V\left(\varphi_{1},\varphi_{2}\right)$*. Then, we have that*

(11) $$\left|\mathrm{Supp}\varphi_{1}\right|+\left|\mathrm{Supp}\varphi_{2}\right|\geq 4.$$

Proof First of all, one can restrict oneself to compactly supported basis functions $\varphi_{1}$ and $\varphi_{2}$ (otherwise, $\left|\mathrm{Supp}\varphi_{1}\right|+\left|\mathrm{Supp}\varphi_{2}\right|=\infty$). If one of the basis function, for instance $\varphi_{2}$, is identically zero, then the cubic spline $\beta^{3}\in V\left(\varphi_{1}\right)$. This means in particular that the basis function $\varphi_{1}$ reproduces polynomials up to degree 3, and its support is therefore at least of size four [16, Theorem 1]. Hence, we again have that $\left|\mathrm{Supp}\varphi_{1}\right|+\left|\mathrm{Supp}\varphi_{2}\right|=\left|\mathrm{Supp}\varphi_{1}\right|\geq 4$. We now assume that $\varphi_{1}$ and $\varphi_{2}$ are not identically 0.

*Step 1.* We show that the extreme points of the supports of $\varphi_{1}$ and $\varphi_{2}$ are integers. For x=a,b,c,d, we set $X\left(\omega \right)=\sum_{k\in Z}x_{k}e^{-j\omega k}$, the 2π-periodic Fourier transform of the sequence x. Condition (10) ensures that X has a periodic continuous third derivative. In the Fourier domain, (8), (9) become

(12) β2^(ω)=(1−e−jω)3(jω)3=A(ω)φ1^(ω)+B(ω)φ2^(ω),

(13) β3^(ω)=(1−e−jω)4(jω)4=C(ω)φ1^(ω)+D(ω)φ2^(ω).

We set $\det\left(\omega \right)=\left(A\left(\omega \right)D\left(\omega \right)-B\left(\omega \right)C\left(\omega \right)\right)$, which is itself a function with continuous third derivative. From (12), (13), we obtain that

(14) det(ω)φ^1(ω)=D(ω)(1−e−jω)3(jω)3−B(ω)(1−e−jω)4(jω)4,

(15) det(ω)φ^2(ω)=−C(ω)(1−e−jω)3(jω)3+A(ω)(1−e−jω)4(jω)4.

From (14), we deduce that, at least when det(ω) does not vanish, we have the relation

(16) (jω)4φ^1(ω)=(jω)F(ω)D(ω)−(1−e−jω)F(ω)B(ω),

where $F\left(\omega \right)=\frac{{\left(1-e^{-j\omega }\right)}^{3}}{\det\left(\omega \right)}$. The strategy of the proof is to show that the function F is continuous and periodic, and that (16) is therefore valid for any ω∈R. We study F in two steps: (i) first, we show that det(ω) does not vanish for ω∉2πZ; and (ii) we then demonstrate that F has a limit at 0 by considering the Taylor expansion of det(ω).

(i) Let us start with the first issue. We show that det(ω)≠0 for ω∉2πZ by contradiction. Let us fix $\omega_{0}\in \left(0,2\pi \right)$ and assume that $\det\left(\omega_{0}\right)=0$. We set $\alpha =\frac{1-e^{-j\omega_{0}}}{j\omega_{0}}$ and $\beta =\frac{1-e^{-j\omega_{0}}}{j\left(\omega_{0}+2\pi \right)}$. Then, α≠0, β≠0, and α≠β, while, by periodicity, $\det\left(\omega_{0}\right)=\det\left(\omega_{0}+2\pi \right)=0$. Hence, (14) for $\omega =\omega_{0}$ and $\left(\omega_{0}+2\pi \right)$ implies that

(17) $$\left(\begin{array}{cc} \alpha^{3} & -\alpha^{4}\\ \beta^{3} & -\beta^{4} \end{array}\right)\left(\begin{array}{c} D\left(\omega_{0}\right)\\ B\left(\omega_{0}\right) \end{array}\right)=\left(\begin{array}{c} 0\\ 0 \end{array}\right).$$

The matrix being invertible (with determinant $\alpha^{3}\beta^{3}\left(\alpha -\beta \right)\neq 0$), we deduce that $D\left(\omega_{0}\right)=B\left(\omega_{0}\right)=0$. Similarly, (15) with $\omega =\omega_{0}$ and $\left(\omega_{0}+2\pi \right)$ implies that $A\left(\omega_{0}\right)=C\left(\omega_{0}\right)=0$. Injecting this in (12) with $\omega =\omega_{0}$, we deduce that β^2(ω0)=α3=0, which contradicts our initial assumption.

(ii) We now study det(ω) around the origin. The function admits a third-order continuous derivative; hence, it can be McLaurin expanded at 0 as

(18) $$\det\left(\omega \right)=\det\left(0\right)+\det^{\left(1\right)}\left(0\right)\omega +\frac{1}{2}\det^{\left(2\right)}\left(0\right)\omega^{2}+\frac{1}{6}\det^{\left(3\right)}\left(0\right)\omega^{3}+o\left(\omega^{3}\right).$$

Assume by contradiction that $\det^{\left(p\right)}\left(0\right)=0$ for p=0,1,2,3. Then, (18) gives that $\det\left(\omega \right)=o\left(\omega^{3}\right)$ around 0. From (14), we remark that

(19) det(ω)(jω)3(1−e−jω)3φ^1(ω)=D(ω)−B(ω)1−e−jωjω.

The function $\frac{\det\left(\omega \right){\left(j\omega \right)}^{3}}{{\left(1-e^{-j\omega }\right)}^{3}}$ vanishes at ω=0 because det does and $\lim_{\omega \to 0}\frac{{\left(j\omega \right)}^{3}}{{\left(1-e^{-j\omega }\right)}^{3}}=1$. Therefore, the left term in (19) vanishes when ω goes to 0. Now, by periodicity, around 2π, we have that $\det\left(\omega \right)=o\left({\left(\omega -2\pi \right)}^{3}\right)$. Hence, $\frac{\det\left(\omega \right)}{{\left(1-e^{-j\omega }\right)}^{3}}=o\left(1\right)$ around 2π and, again, the left term in (19) is also vanishing when ω goes to 2π.

We deduce that the right term in (19) vanishes for both ω=0 and ω=2π. In other terms, we have that

(20) 0=D(0)−B(0)=D(2π)−0

and, since D(2π)=D(0) by periodicity, this implies that D(0)=B(0)=0. A similar reasoning shows that A(0)=C(0)=0. From (12) with ω=0, we obtain that β^3(0)=0, which is false.

As a consequence, at least one of the derivative of the McLaurin expansion (18) is nonzero, from which we easily deduce that $F\left(\omega \right)=\frac{{\left(1-e^{-j\omega }\right)}^{3}}{\det\left(\omega \right)}$ has a limit (possibly 0) at the origin. The function F is well-defined and continuous for ω∉2πZ, continuously extendable at 0, and is therefore a continuous periodic function.

At this stage, we obtained that (16) is valid for any ω∈R. The functions F(ω)D(ω) and $\left(1-e^{-j\omega }\right)F\left(\omega \right)B\left(\omega \right)$ are 2π-periodic, hence their inverse Fourier transforms are sums of Dirac impulses located at the integers. It means in particular that we have, in the time domain, that

(21) $$\varphi_{1}^{\left(4\right)}\left(t\right)=\underset{k\in Z}{\sum }\left(y_{k}\delta \left(t-k\right)+z_{k}\delta^{\prime }\left(t-k\right)\right),$$

where y and z are the Fourier sequences of $\left(1-e^{-j\omega }\right)F\left(\omega \right)B\left(\omega \right)$ and F(ω)D(ω), respectively. Since $\varphi_{1}^{\left(4\right)}$ is compactly supported, like $\varphi_{1}$, only finitely many $y_{k}$ and $z_{k}$ are non-zero. Then, $\varphi_{1}$ is a compactly supported function whose fourth derivative has a support with integer extreme points (due to (21)), and therefore has a support with integer extreme points., too The same reasoning applies for $\varphi_{2}$, which concludes this part of the proof.

*Step 2.* We know that the supports of $\varphi_{1}$ and $\varphi_{2}$ are of the form [a,b] with a<b, a,b∈Z. By contradiction, we assume that $\left|\mathrm{Supp}\varphi_{1}\right|+\left|\mathrm{Supp}\varphi_{2}\right|<4$. Then, one of the two basis functions has a support of size one, for instance $\varphi_{1}$. We also assume without loss of generality that $\mathrm{Supp}\varphi_{1}=\left[0,1\right]$, implying that only $y_{0},y_{1},z_{0},z_{1}$ are possibly nonzero in (21). Going back to the Fourier domain, one obtains that

(22) (jω)4φ^1(ω)=y0+y1e−jω+jω(z0+z1e−jω).

The function $\varphi_{1}$ is compactly supported. Its Fourier transform is hence infinitely smooth, and we can do the McLaurin expansion of both sides in (22) up to order 3. This gives

(23) $$o\left(\omega^{3}\right)=\left(y_{0}+y_{1}\right)+j\omega \left(-y_{1}+z_{0}+z_{1}\right)+\omega^{2}\left(-y_{1}∕2+z_{1}\right)+j\omega^{3}\left(y_{1}∕6-z_{1}∕2\right)+o\left(\omega^{3}\right).$$

In particular, we obtain the relations

(24) $$y_{0}+y_{1}=z_{0}+z_{1}-y_{1}=z_{1}-\frac{y_{1}}{2}=\frac{y_{1}}{6}-\frac{z_{1}}{2}=0.$$

This imposes that $y_{0}=y_{1}=z_{0}=z_{1}=0$, which is absurd. Finally, it shows that $\left|\mathrm{Supp}\varphi_{1}\right|+\left|\mathrm{Supp}\varphi_{2}\right|\geq 4$, as expected.  □

Condition (10) plays an important role in our proof by imposing some regularity in the Fourier domain. In practice, one even expects that compactly supported basis functions can generate the B-splines $\beta^{2}$ and $\beta^{3}$ with finitely many coefficients, in which case (10) is automatically satisfied. However, we believe that (10) can be relaxed up to some extent. From Theorem 3, we easily deduce that Hermite splines have the minimal-support property.

**Corollary** 4 *The Hermite splines* $\left(\phi_{1},\phi_{2}\right)$ *are of minimal support among the pairs of functions that are able to reproduce both quadratic and cubic B-splines with reproduction sequences satisfying* (10)*.*

Proof From [13, Appendix A], we know that Hermite splines can reproduce both quadratic and cubic B-splines, hence $\beta^{2}$ and $\beta^{3}$ are in $V\left(\phi_{1},\phi_{2}\right)$ with compactly supported reproduction sequences obviously satisfying (10). The supports of $\phi_{1}$ and $\phi_{2}$ are of size two, which implies that $\left|\mathrm{Supp}\phi_{1}\right|+\left|\mathrm{Supp}\phi_{2}\right|=4$. Finally, the pair $\left(\phi_{1},\phi_{2}\right)$ is maximally localized due to (11).  □

It is worth noting that the supports of the pair of Hermite splines jointly has the same size as the B-spline $\beta^{3}$. However, $\beta^{3}$ alone has lesser reproduction properties. Being of class $C^{2}$, it is in particular unable to reproduce the quadratic spline $\beta^{2}$, which only has $C^{1}$ transitions at the integers. The simplest way of reproducing $\beta^{2},\beta^{3}$ is to consider the basis pair $\left(\beta^{2},\beta^{3}\right)$ itself, which is not maximally localized since the sum of the supports is 7. An important additional remark is that two functions are needed to reproduce both cubic and quadratic spline, as formalized in Proposition 5.

Proposition 5 *There exists no single function* $\varphi \in L_{2}\left(R\right)$ *that is able to reproduce* $\beta^{2}$ *and* $\beta^{3}$ *with summable reproduction sequences.*

Proof By contradiction, let us assume that there exists φ such that $\beta^{2}=\sum_{k\in Z}a_{k}\varphi \left(\cdot -k\right)$ and $\beta^{3}=\sum_{k\in Z}b_{k}\varphi \left(\cdot -k\right)$ with $a,b\in \ell_{1}\left(Z\right)$. Then, the Fourier transforms $A\left(e^{j\omega }\right)$ and $B\left(e^{j\omega }\right)$ are continuous 2π-periodic functions. In the Fourier domain, we have that

(25) 1−e−jωjω3=A(ejω)φ^(ω),1−e−jωjω4=B(ejω)φ^(ω).

Set $\omega_{0}\in \left(0,2\pi \right)$ and $\omega_{1}=\omega_{0}+2\pi$. The relation (25) imposes that $A\left(e^{j\omega_{i}}\right)$, $B\left(e^{j\omega_{i}}\right)$, and φ^(ωi) are non-zero for i=1,2, and

(26) 1−e−jωijωiA(ejωi)φ^(ωi)=B(ejωi)φ^(ωi).

After simplifications, we deduce that

(27) $$j\omega_{i}=\frac{\left(1-e^{-j\omega_{i}}\right)A\left(e^{j\omega_{i}}\right)}{B\left(e^{j\omega_{i}}\right)}.$$

The right term in (27) is equal for $\omega_{0}$ and $\omega_{1}$ by periodicity, while the left term is not. This contradicts our initial assumption and implies Proposition 5.  □

## 3 Approximation properties of Hermite splines

Existing approaches have been proposed to characterize the behavior of the approximation error for single [19] and multi-generators [20]. They however assume technical conditions that the Hermite interpolation does not satisfy. We therefore formulate new theoretical tools to quantify the asymptotic constant of the error of approximation of this scheme. Since functions are estimated with derivative samples in the Hermite case, we also propose to investigate the approximation error on the first derivative. In our setting, other existing approximation schemes can be considered as well, allowing us to relate the excellent approximation properties of Hermite splines to other schemes such as cubic B-splines and interlaced derivative sampling.

### 3.1 Generalized sampling and error of approximation

The approximation of a continuously defined signal from a collection of its samples in a generalized-sampling scheme relies on two ingredients: some basis functions $\varphi =\left(\varphi_{1},\ldots ,\varphi_{N}\right)\in {\left(L_{2}\left(R\right)\right)}^{N}$ forming a Riesz basis in the sense of (6), and some *sampling functions* $\overset{\sim }{\varphi }=\left({\overset{\sim }{\varphi }}_{1},\ldots ,{\overset{\sim }{\varphi }}_{N}\right)$ that are rapidly decaying generalized functions. The sampling functions include rapidly decaying $L_{2}$-integrable functions together with the Dirac impulse δ, its derivative, and their shifts. We recall that an $L_{2}$ function is *rapidly decaying* if it decays faster than the inverse of any polynomial at infinity. A generalized function in $S^{\prime }\left(R\right)$ is rapidly decaying if its convolution with any infinitely differentiable and rapidly decaying function is a rapidly decaying function [38]. In particular, a rapidly decaying (generalized) function has an infinitely differentiable Fourier transform, a property which we shall rely on thereafter. The term *generalized* is motivated by the fact that sampling functions allow one to access more than just the values of the signal at the sampling points [39].

The set of pairs of basis and sampling functions fully characterizes an approximation scheme. Moreover, because we aim at a fair comparison, the quantity of information per unit of time ought to be equal among the considered schemes. This requires us to slightly adapt the definition of V(φ) given in (5). From now, we shall consider

(28) $$W\left(\varphi \right)=\left\{\overset{N}{\underset{i=1}{\sum }}\underset{k\in Z}{\sum }c_{i}\left[k\right]\varphi_{i}\left(\cdot -Nk\right):c=\left(c_{1},\ldots ,c_{N}\right)\in {\left(\ell_{2}\left(Z\right)\right)}^{N}\right\}.$$

The added parameter N ensures that, for any N≥1, there is on average a single degree of freedom on each interval of size one. The sampling and reconstruction problem is then formally defined as follows: the function f is reconstructed by its approximation $\overset{\sim }{f}$ associated to the basis functions φ and for the sampling functions $\overset{\sim }{\varphi }$, defined as

(29) $$\overset{\sim }{f}=\overset{N}{\underset{i=1}{\sum }}\underset{k\in Z}{\sum }\langle f,{\overset{\sim }{\varphi }}_{i}\left(\cdot -Nk\right)\rangle \varphi_{i}\left(\cdot -Nk\right)\in W\left(\varphi \right).$$

We denote by $Q_{\varphi }^{\overset{\sim }{\varphi }}$ the operator such that $Q_{\varphi }^{\overset{\sim }{\varphi }}f=\overset{\sim }{f}$. Hermite-spline approximation thus corresponds to N=2 with basis functions $\varphi_{1}\left(t\right)=\phi_{1}\left(\frac{t}{2}\right)$ and $\varphi_{2}\left(t\right)=2\phi_{2}\left(\frac{t}{2}\right)$, and sampling functions ${\overset{\sim }{\varphi }}_{1}\left(t\right)=\delta \left(t\right)$ and ${\overset{\sim }{\varphi }}_{2}\left(t\right)=-\delta^{\prime }\left(t\right)$.

The best approximation of a given scheme is obtained when the pairs of sampling and basis functions are properly chosen such that $\overset{\sim }{f}$ is the orthogonal projection of f onto W(φ). This implies the imposition of a particular condition [20] on the sampling functions $\overset{\sim }{\varphi }$, namely, that

(30) φ~=φ1,d⋮φN,d=F−1{Gφ(⋅)−1φ^},

where $G_{\varphi }$ is the Gram matrix of size $\left(N\times N\right)$ associated to φ, given for ω∈R by

(31) Gφ(ω)=∑k∈Zφ^(ω+2kπ)φ^∗T(ω+2kπ).

This particular collection of sampling functions is called the *dual functions* associated to φ and is denoted by $\varphi_{d}$. Note that $G_{\varphi }\left(\omega \right)$ is invertible for every ω∈R and, therefore, (30) is meaningful because (6) is equivalent to $0<A\leq \lambda_{\min}\left(\omega \right)\leq \lambda_{\max}\left(\omega \right)\leq B<\infty$, where $\lambda_{\min}\left(\omega \right)$ ($\lambda_{\max}\left(\omega \right)$, respectively) is the minimum (maximum, respectively) eigenvalue of $G_{\varphi }\left(\omega \right)$ [40]. When $\overset{\sim }{\varphi }$ is defined as (30), the operator $Q_{\varphi }^{\overset{\sim }{\varphi }}$ is the orthogonal projector over W(φ), denoted as $P_{\varphi }=Q_{\varphi }^{\varphi_{d}}$. In this situation, (29) is reformulated as

(32) $$\overset{\sim }{f}\left(t\right)=P_{\varphi }f=\overset{N}{\underset{i=1}{\sum }}\underset{k\in Z}{\sum }\langle f,\varphi_{i,d}\left(\cdot -Nk\right)\rangle \varphi_{i}\left(\cdot -Nk\right).$$

To simplify the notation, we shall write $P=P_{\varphi }$ and $Q=Q_{\varphi }^{\overset{\sim }{\varphi }}$ thereafter. The quality of the approximation is then evaluated in terms of the error of approximation which is expressed as

(33) $$E_{\varphi }^{\overset{\sim }{\varphi }}\left(f\right)=\|f-\overset{\sim }{f}{\|}_{L_{2}}=\|f-Qf{\|}_{L_{2}}.$$

When $\overset{\sim }{f}=Pf$, the error is denoted as $E_{\varphi }\left(f\right)$. A direct implication is that $E_{\varphi }\left(f\right)=E_{\varphi }^{\varphi_{d}}\left(f\right)\leq E_{\varphi }^{\overset{\sim }{\varphi }}\left(f\right)$, reaching the equality if and only if $\overset{\sim }{\varphi }=\varphi_{d}$.

Up to now, we considered approximation schemes with (generalized) samples taken on the integer grid, which corresponds to the sampling step T=1. This parameter affects the coarseness of the approximation: when T→0, the error is expected to vanish. For T>0, the approximation space (28) becomes

(34) $$W_{T}\left(\varphi \right)=\left\{\overset{N}{\underset{i=1}{\sum }}\underset{k\in Z}{\sum }c_{i}\left[k\right]\varphi_{i}\left(\frac{\cdot }{T}-Nk\right)\;:\;c\in {\left(\ell_{2}\left(Z\right)\right)}^{N}\right\}$$

and the approximation of f is given by

(35) $${\overset{\sim }{f}}_{T}=Q_{T}f=\overset{N}{\underset{i=1}{\sum }}\underset{k\in Z}{\sum }\langle f,\frac{1}{T}{\overset{\sim }{\varphi }}_{i}\left(\frac{\cdot }{T}-Nk\right)\rangle \varphi_{i}\left(\frac{\cdot }{T}-Nk\right),$$

with resulting error

(36) $$E_{\varphi }^{\overset{\sim }{\varphi }}\left(f,T\right)=\|f-Q_{T}f{\|}_{L_{2}}.$$

The orthogonal projector (32) and its associated error are easily reformulated accordingly.

A number of hypotheses on the basis functions φ and the sampling functions $\overset{\sim }{\varphi }$ have to be met in order to study the errors $E_{\varphi }\left(f,T\right)$ and $E_{\varphi }^{\overset{\sim }{\varphi }}\left(f,T\right)$ in terms of rate of decay and asymptotic constant. The first one is the Riesz-basis condition (6), which ensures a unique and stable representation. The second one is the order of the basis functions. We shall consider basis functions φ of a given order in the sense of Definition 1.

When it is met, the decrease of the optimal error $E_{\varphi }\left(f,T\right)$ is bounded from above by $T^{L}$ [20]. The last important condition is that the sampling and basis functions are quasi-biorthonormal of order L.

**Definition** 6 Two families of bases φ and sampling functions $\overset{\sim }{\varphi }$ are *quasi-biorthonormal of order* L if the basis functions are of order L and, for the dual function $\varphi_{d}$ given by (30) and all ℓ=0,…,(L−1), we have that

(37) $$\int_{R}t^{\ell }\overset{\sim }{\varphi }\left(t\right)dt=\int_{R}t^{\ell }\varphi_{d}\left(t\right)dt.$$

It is worth noting that (37) is a slight abuse of notation, since the ${\overset{\sim }{\varphi }}_{i}$ are not necessarily defined pointwise. However, they are assumed to be rapidly decaying generalized functions and can therefore be taken against a slowly growing smooth function such as $t\mapsto t^{\ell }$. The right term in (37) is well-defined due to the condition $\int_{R}{\left(1+\left|t\right|\right)}^{L}\left|\varphi_{n}\left(t\right)\right|dt<\infty$ in Definition 1, as discussed in [20].

The quasi-biorthonormality ensures that the decrease of the error $E_{\varphi }^{\overset{\sim }{\varphi }}\left(f,T\right)$ is also bounded by $T^{L}$ [20]. Finally, the rate of decay of the error of approximation being under control in all generality, the asymptotic constant can be obtained as

(38) $$C_{\varphi }^{\overset{\sim }{\varphi }}\left(f\right)=\underset{T\to 0}{\lim}T^{-L}E_{\varphi }^{\overset{\sim }{\varphi }}\left(f,T\right)$$

which, in practice, can be computed relying on a Fourier-domain approximation-error kernel. For more details, we refer the interested reader to [41], [42], [43] and references therein.

### 3.2 Approximation constants of irregular sampling schemes

Our goal in this section is to extend the main results of [20] so as to include Hermite-spline approximations. The original framework is indeed restricted to sampling functions $\overset{\sim }{\varphi }$ with bounded Fourier transforms. While this allows one to consider the case of interpolation, it excludes Hermite-spline approximations since the Fourier transform jω of $\delta^{\prime }$ is unbounded.

In what follows, it will be useful to consider Sobolev spaces of integer order N. For N≥0, we define $W_{2}^{N}\left(R\right)$ as the space of functions f such that

(39) ∫R|f^(ω)|2(1+ω2)Ndω<∞.

For N=0, this corresponds to the space $L_{2}\left(R\right)$. In general, $f\in W_{2}^{N}\left(R\right)$ if and only if $f,\ldots ,f^{\left(N\right)}\in L_{2}\left(R\right)$. For technical reasons, we also consider Sobolev spaces of fractional order γ≥0, for which we simply replace the integer N by a nonnegative real number γ in (39). Fractional Sobolev spaces are intimately connected to fractional derivatives in the following sense: the fractional derivative of order γ≥0 is defined in the Fourier domain by

(40) F{f(γ)}(ω)=(jω)γf^(ω)

for any $f\in W_{2}^{\gamma }\left(R\right)$. By definition of the Sobolev space, one has that $f^{\left(\gamma \right)}\in L_{2}\left(R\right)$ as soon as $f\in W_{2}^{\gamma }\left(R\right)$. In general, we have the Parseval-type relation ‖f(γ)‖L22=∫R|f^(ω)|2|ω|2γdω.

Our development follows the key contributions of [20]. We therefore only detail the adaptations that are required in our case. We start with a brief summary of the general approach, which is common to many works of approximation theory in shift-invariant spaces. We first introduce the kernels associated to the functions $\varphi ,\overset{\sim }{\varphi }$ as

(41) Emin(ω)=1+φ^∗T(ω)Gφ−1(ω)φ^(ω),

(42) Eres(ω)=(φ~^−φd^)∗T(ω)Gφ(ω)(φ~^−φd^)(ω),

(43) $$E\left(\omega \right)=E_{\min}\left(\omega \right)+E_{\mathrm{res}}\left(\omega \right),$$

where $G_{\varphi }$ is the Gram matrix (31). The kernel $E_{\min}$ relates to the *minimum-error* case achieved using the orthogonal projector (*i.e.*, $\overset{\sim }{\varphi }=\varphi_{d}$), and $E_{\mathrm{res}}$ to the *residual error* that arise when using sampling functions that differ from the dual functions. We furthermore note that $E_{\mathrm{res}}\left(\omega \right)=0$ when $\overset{\sim }{\varphi }=\varphi_{d}$, as expected. The key ideas of relying on E are as follows.

• The kernel E measures the approximation power of a reasonable approximation scheme $\left(\varphi ,\overset{\sim }{\varphi }\right)$, in the sense that ‖f−QTf‖L2≈∫R|f^(ω)|2E(Tω)dω1∕2 for small T>0.
• The precise behavior of $\|f-Q_{T}f{\|}_{L_{2}}$ is then deduced from that of E around the origin. It depends on the approximation order L of the scheme and, typically, behaves like $CT^{L}$. The constant C depends on the function f to approximate and on the scheme $\left(\varphi ,\overset{\sim }{\varphi }\right)$ via the Taylor expansion of E.

We give a precise meaning to these two points in Proposition 8, Proposition 9. Before that, we recall an important lemma taken from [20] which will play a fundamental role in our proofs.

**Lemma** 7 *Let* $f\in W_{2}^{L+1}\left(R\right)$*. For* k≥0*, we set* f^k(ω)=f^(ω)1k∕T≤|ω|<(k+1)∕T*. Then, the following relations hold:*

(44) f^=∑k≥0f^k;

(45) ‖fk−QTfk‖L22=∫R|f^k(ω)|2E(Tω)dω for k≥0; and

(46) $$|\|f-Q_{T}f{\|}_{L_{2}}-\|f_{0}-Q_{T}f_{0}{\|}_{L_{2}}|\leq \underset{k>0}{\sum }\|f_{k}-Q_{T}f_{k}{\|}_{L_{2}}.$$

Equality (44) is obvious. The two next relations come from [20, Theorem 1]; we have simply reformulated the results with our notation. First, it is proven (see [20, Theorem 1]) that ‖f−QTf‖L22=∑k∈Z∫R|f^k(ω)|2E(Tω)dω as soon as f^(ω)f^(ω−n∕T)=0 for any ω∈R and n∈Z, a condition that is satisfied by the $f_{k}$ by construction, giving (45). Moreover, the inequality (46) appears in the proof of [20, Theorem 1] (see (63)).

Proposition 8 *Let* $\left(\varphi ,\overset{\sim }{\varphi }\right)$ *be a set of* N *basis and sampling functions that are quasi-biorthonormal and provide an approximation scheme of order*L*. We assume moreover that the kernel* E *given by* (43) *satisfies*

(47) $$\left|E\left(\omega \right)\right|\leq C^{2}\max\left(1,{\left|\omega \right|}^{2p}\right)$$

*for some* C>0*, some integer* 0≤p≤L*, and every* ω∈R*. Then, for every* $f\in W_{2}^{L+1}\left(R\right)$*, we have that*

(48) ‖f−QTf‖L2=∫R|f^(ω)|2E(Tω)dω1∕2+O(TL+1).

The case p=0 corresponds to a bounded E and can be found in [20, Theorem 1].

Proof We recall that f^k(ω) is defined as f^k(ω)=f^(ω)1k∕T≤|ω|<(k+1)∕T, and that fk=F−1{f^k}. Then, we have that

(49) |‖f−QTf‖L2−∫R|f^(ω)|2E(Tω)dω1∕2|≤|‖f−QTf‖L2−‖f0−QTf0‖L2|+|‖f0−QTf0‖L2−∫R|f^(ω)|2E(Tω)dω1∕2|≤(i)∑k>0‖fk−QTfk‖L2+|‖f0−QTf0‖L2−∫R|f^(ω)|2E(Tω)dω1∕2|=(I)+(II),

where we used (46) in (i). As a consequence, (48) follows if one shows that the two terms (I) and (II) in (49) are $O\left(T^{L+1}\right)$. Using (45), we have that

(50) ‖fk−QTfk‖L22=∫k∕T<|ω|≤(k+1)∕T|f^k(ω)|2E(Tω)dω=∫k∕T<|ω|≤(k+1)∕T|f^k(ω)|2|ω|2(L+1)T2p|ω|2(L−p+1)E(Tω)T2p|ω|2pdω≤C2T2p(k∕T)2(L−p+1)∫R|f^k(ω)|2|ω|2(L+1)dω=C2T2(L+1)k2(L−p+1)‖fk(L+1)‖L22,

where the inequality is due to |ω|≥k∕T over the domain and to (47), which implies that $C^{2}\geq \sup_{\omega \in R}E\left(\omega \right)∕\max\left(1,{\left|\omega \right|}^{p}\right)\geq \sup_{\left|\omega \right|\geq 1}E\left(\omega \right)∕{\left|\omega \right|}^{p}$. Summing over k>0, we deduce that

(51) $$\left(I\right)\leq CT^{L+1}\underset{k>0}{\sum }\frac{1}{k^{L-p+1}}\|f_{k}^{\left(L+1\right)}{\|}_{L_{2}}\leq CT^{L+1}{\left(\underset{k>0}{\sum }\frac{1}{k^{2\left(L-p+1\right)}}\right)}^{1∕2}{\left(\underset{k>0}{\sum }\|f_{k}^{\left(L+1\right)}{\|}_{L_{2}}^{2}\right)}^{1∕2}=CT^{L+1}\sqrt{\zeta \left(2\left(L-p+1\right)\right)}\|{\left(f-f_{0}\right)}^{\left(L+1\right)}{\|}_{L_{2}}=O\left(T^{L+1}\right),$$

where the second inequality is derived from Cauchy–Schwarz, and ζ is the Riemann zeta function. For the second term, we remark that, again due to [20, (27)], ‖f0−QTf0‖L22=∫|ω|≤1∕T|f^(ω)|2E(Tω)dω. Therefore, using the relation $\left|\|g{\|}_{L_{2}}-\|h{\|}_{L_{2}}\right|\leq \|g-h{\|}_{L_{2}}$ (a consequence of the Minkowski inequality) we deduce that

(52) (II)≤∫|ω|>1∕T|f^(ω)|2E(Tω)dω1∕2=∫|ω|>1∕T|f^(ω)|2|ω|2(L+1)T2p|ω|2(L−p+1)E(Tω)T2p|ω|2pdω1∕2≤CTpTL−p+1∫|ω|>1∕T|f^(ω)|2|ω|2(L+1)dω1∕2=CTL+1‖(f−f0)(L+1)‖L2=O(TL+1),

where the inequality once more follows from |ω|≥1∕T over the domain and from our assumption (47). Combining (51), (52) in (49) completes the proof. □

Our next result connects the expression ∫R|f^(ω)|2E(Tw)dω to the McLaurin expansion of E.

Proposition 9 *Let* $\left(\varphi ,\overset{\sim }{\varphi }\right)$ *be a set of* N *basis and sampling functions that are quasi-biorthonormal and provide an approximation scheme of order*L*. We assume moreover that the kernel* E *given by* (43) *is* (2L+1)*-times continuously differentiable and satisfies*

(53) $$\left|E^{\left(2L+1\right)}\left(\omega \right)\right|\leq C^{2}\max\left(1,{\left|\omega \right|}^{2p}\right)$$

*for some* C>0*, some integer* 0≤p≤L*, and every* ω∈R*. Then, for every* $f\in W_{2}^{L+p+1∕2}\left(R\right)$*, we have that*

(54) ∫R|f^(ω)|2E(Tw)dω=E(2L)(0)(2L)!‖f(L)‖L22T2L+O(T2L+1).

Proof The kernel E being symmetric, we deduce that $E^{\left(2k+1\right)}\left(0\right)=0$ for k=0,…,(L−1). Moreover, the condition of $\left(\varphi ,\overset{\sim }{\varphi }\right)$ ensures that $E^{\left(2k\right)}\left(0\right)=0$ for k=0,…,(L−1), together with $E^{\left(2L\right)}\left(0\right)\neq 0$. Therefore, E being (2L+1)-times continuously differentiable, its McLaurin expansion is given by

(55) $$E\left(\omega \right)=\frac{E^{\left(2L\right)}\left(0\right)}{\left(2L\right)!}\omega^{2L}+\frac{E^{\left(2L+1\right)}\left(\omega \theta \right)}{\left(2L+1\right)!}w^{2L+1},$$

with θ=θ(ω)∈(0,1). Using (55), we deduce that

(56) ∫R|f^(ω)|2E(Tw)dω=∫R|f^(ω)|2E(2L)(0)(2L)!T2Lω2Ldω+T2L+1(2L+1)!∫R|f^(ω)|2ω2L+1E(2L+1)(ωTθ(ωT))dω=E(2L)(0)(2L)!‖f(L)‖L22T2L+T2L+1(2L+1)!∫R|f^(ω)|2ω2L+1E(2L+1)(ωTθ(ωT))dω.

Due to (53) and 0<θ(ωT)<1,

(57) $$E^{\left(2L+1\right)}\left(\omega T\theta \left(\omega T\right)\right)\leq C^{2}\max\left(1,{\left|\omega \right|}^{2p}T^{2p}\right)\leq C^{2}\left(1+{\left|\omega \right|}^{2p}T^{2p}\right),$$

from which we deduce that ∫R|f^(ω)|2ω2L+1E(2L+1)(ωTθ(ωT))dω≤C2(‖f(L+1∕2)‖L22+T2p‖f(L+p+1∕2)‖L22). Note that we refer here to fractional derivatives, as defined in (40). Injecting this to (56) implies that

(58) ∫R|f^(ω)|2E(Tw)dω−E(2L)(0)(2L)!‖f(L)‖L22T2L=C2T2L+1(2L+1)!(‖f(L+1∕2)‖L22+T2p‖f(L+p+1∕2)‖L22)=O(T2L+1),

which concludes the proof. □

Finally, we conclude with an extension of [20, Theorem 4] to sampling functions that are not necessarily bounded in the Fourier domain.

**Theorem** 10 *We consider an approximation scheme* $\left(\varphi ,\overset{\sim }{\varphi }\right)$ *with* N *basis and sampling functions such that*

• *the basis functions are rapidly decaying* $L_{2}$ *functions such that the family* ${\left\{\varphi_{i}\left(\cdot -Nk\right)\right\}}_{i=1\ldots N,k\in Z}$ *is a Riesz basis in the sense of* (6)*, with approximation power of order* L≥1 *(see* Definition 1*);*
• *the sampling functions are rapidly decaying generalized functions such that* $\left(\varphi ,\overset{\sim }{\varphi }\right)$ *is quasi-biorthonormal of order* L *(see* Definition 6*);*
• *there exists an integer* 0≤p≤L *such that, for any* ω∈R*, any* 0≤k≤L*, and any* 1≤i≤N*,*
  (59) |φ~^i(k)(ω)|≤Cmax(1,|ω|p).

*Then, for any* $f\in W^{L+\max\left(p+1∕2,1\right)}\left(R\right)$*, we have that*

(60) $$\|f-Q_{T}f{\|}_{L_{2}}\underset{T\to 0}{∼}\sqrt{\frac{E^{\left(2L\right)}\left(0\right)}{\left(2L\right)!}}\|f^{\left(L\right)}{\|}_{L_{2}}T^{L}.$$

The two first conditions in Theorem 10 are necessary to ensure that the approximation scheme has an approximation power of order L. The last condition allows us to show that such an approximation power is attained with restricted condition on the sampling functions having a possibly unbounded Fourier transform.

Proof We prove that the conditions of Theorem 10 imply that we fulfill the hypotheses of both Proposition 8, Proposition 9.

Since $\varphi ,\overset{\sim }{\varphi }$ are rapidly decaying (generalized) functions, their Fourier transforms φ^ and φ~^ are infinitely differentiable. The same holds true for φ^d=Gφ−1φ^ since $G_{\varphi }$ is a matrix-valued infinitely differentiable function with infinitely differentiable inverse $G_{\varphi }^{-1}$ due to the Riesz basis condition. This implies that E is infinitely differentiable and therefore (2L+1)-times continuously differentiable.

The basis functions are rapidly decaying, hence in $L_{1}\left(R\right)$, which implies that φ^ is bounded. The Riesz-basis condition then implies that both $G_{\varphi }$ and its inverse are bounded as functions of ω. It then follows that $E_{\min}\left(\omega \right)$ is bounded, while $E_{\mathrm{res}}\left(\omega \right)$ is dominated by ‖(φ~^−φ^d)(ω)‖, itself being dominated by $\max\left(1,{\left|\omega \right|}^{2p}\right)$ due to (59). It finally implies (47) for some constant C>0. Similarly, for k≤L, the function φ^(k) is bounded, being the Fourier transform of $t\mapsto t^{k}\varphi \left(t\right)$, which is in $L_{1}\left(R\right)$ due to the rapid decay of φ. Again, this property is transferred to the derivative of $G_{\varphi }$ and its inverse. Then, exploiting the Leibnitz rule, one shows that $E_{\min}^{\left(2L+1\right)}$ is bounded, while $E^{\left(2L+1\right)}$ is a sum of products made of bounded terms and of two terms of the form φ~^i(ω), which are controlled by (59). Putting things together, we deduce (53) for some constant C>0.

Finally, the hypotheses of Proposition 8, Proposition 9 are satisfied, implying (48), (54), together with

(61) $$\|f-Q_{T}f{\|}_{L_{2}}=\sqrt{\frac{E^{\left(2L\right)}\left(0\right)}{\left(2L\right)!}}\|f^{\left(L\right)}{\|}_{L_{2}}T^{L}+O\left(T^{L+1∕2}\right).$$

This proves (60). □

Note that [20, Theorem 4] corresponds to Theorem 10 with p=1, and with less restrictive assumptions on φ and $\overset{\sim }{\varphi }$ (essentially, the rapid decay is replaced by a polynomial decay adapted to L). The condition $f\in W^{L+\max\left(p+1∕2,1\right)}\left(R\right)$ reflects the cost of covering the scenario of possibly irregular sampling functions with unbounded Fourier transforms.

### 3.3 Approximation properties of Hermite splines

We can now evaluate the approximation error on f in different frameworks, including the Hermite scheme. In our analysis, for $\left(\varphi ,\overset{\sim }{\varphi }\right)$ being fixed, we also quantify the error on the derivative $f^{\prime }$ when we approximate it by ${\left(Q_{T}f\right)}^{\prime }$. We therefore study the quantities $\|f-Q_{T}f{\|}_{L_{2}}$ and $\|f^{\prime }-{\left(Q_{T}f\right)}^{\prime }{\|}_{L_{2}}$. Knowing the order of approximation L≥1, the quality of the approximation is quantified by the two asymptotic constants

(62) $$C_{\varphi }^{\overset{\sim }{\varphi }}=\left(\begin{array}{c} C_{\varphi ,1}^{\overset{\sim }{\varphi }}\\ C_{\varphi ,2}^{\overset{\sim }{\varphi }} \end{array}\right)=\left(\begin{array}{c} \underset{T\to 0}{\lim}T^{-L}\|f^{\left(L\right)}{\|}_{L_{2}}^{-1}\|f-Q_{T}f{\|}_{L_{2}}\\ \underset{T\to 0}{\lim}T^{-\left(L-1\right)}\|f^{\left(L-1\right)}{\|}_{L_{2}}^{-1}\|f^{\prime }-{\left(Q_{T}f\right)}^{\prime }{\|}_{L_{2}} \end{array}\right).$$

The asymptotic constant $C_{\varphi ,2}^{\overset{\sim }{\varphi }}$ can be computed with the same tools as $C_{\varphi ,1}^{\overset{\sim }{\varphi }}$. Using integration by parts, we indeed have that

(63) $${\left(Q_{T}f\right)}^{\prime }=\frac{1}{T}\overset{N}{\underset{i=1}{\sum }}\underset{k\in Z}{\sum }\langle f,\frac{1}{T}{\overset{\sim }{\varphi }}_{i}\left(\frac{\cdot }{T}-Nk\right)\rangle \varphi_{i}^{\prime }\left(\frac{\cdot }{T}-Nk\right)$$

(64) $$=\frac{1}{T}\overset{N}{\underset{i=1}{\sum }}\underset{k\in Z}{\sum }\langle f^{\prime },\frac{1}{T}{\overset{\sim }{\varphi }}_{i,\mathrm{int}}\left(\frac{\cdot }{T}-Nk\right)\rangle \varphi_{i}^{\prime }\left(\frac{\cdot }{T}-Nk\right),$$

where the new sampling functions are best defined in the Fourier domain as

(65) φ~i,int^(ω)=−1jωφ~i^(ω).

As one power of T gets lost in the differentiation process, the rate of decay of the error on $f^{\prime }$ is (L−1). Note that the apparent singularity in (65) around 0 is counterbalanced in the analysis since one only approximates functions $f^{\prime }$ that are derivatives functions, hence, for which f′^(ω)=jωf^(ω).

In the Hermite framework, for which N=2, sampling functions are taken as ${\overset{\sim }{\varphi }}_{1}=\delta$ and ${\overset{\sim }{\varphi }}_{2}=-\delta^{\prime }$ and basis functions as (2) and (3). For comparison purpose, we also consider two relevant schemes that fit our analysis framework: classical cubic B-splines and interlaced derivative sampling. Cubic B-spline approximation corresponds to N=1, with $\overset{\sim }{\varphi }=\sum_{k\in Z}{\left(b^{3}\right)}^{-1}\left[k\right]\delta \left(\cdot -k\right)$, where ${\left(b^{3}\right)}^{-1}$ is the direct B-spline filter and $\varphi =\beta^{3}$ the cubic B-spline [37]. Interlaced derivative sampling can be defined in the more general framework of generalized sampling without band-limited constraints [41]. In this setting, N=2 and the sampling functions correspond to ${\overset{\sim }{\varphi }}_{1}=\delta$ and ${\overset{\sim }{\varphi }}_{2}=-\delta^{\prime }\left(\cdot -\frac{1}{2}\right)$. The basis functions are constructed from the cubic B-spline to allow for a fair comparison and are given by

(66) φ^1(ω)=3e−2jω−1+ejω42ω4,

(67) φ^2(ω)=e−2jω−1+ejω41+ejω−4+ejω2−2e2jωω4

in the Fourier domain. A comprehensive description of their derivation is provided in [43]. In particular, the family $\left(\varphi_{1},\varphi_{2}\right)$ is known to be of order 4. It is worth noting that the sampling functions $\left({\overset{\sim }{\varphi }}_{1},{\overset{\sim }{\varphi }}_{2}\right)=\left(\delta ,-\delta^{\prime }\left(\cdot -1∕2\right)\right)$ do not correspond to the dual functions in these frameworks. This is easily motivated by practical considerations: the sampling process is, in practice, implemented with digital filtering, which excludes dual functions due to their continuous nature. The dual functions can, however, still be constructed from the Gram matrix following (30) so as to estimate the optimal approximation error.

We now reveal the approximation power of these three different schemes. The results are known for the cubic B-splines [19] and are included for comparison purposes. For Hermite approximation and interlaced sampling, they are deduced from Theorem 10 and are not included in the multi-generator framework [20], whose hypotheses exclude the use of derivative samples.

Proposition 11 *Let* $\left(\varphi ,\overset{\sim }{\varphi }\right)$ *be one of the three approximation schemes considered above. Then, we have that*

(68) $$\|f-Q_{T}f{\|}_{L_{2}}\underset{T\to 0}{∼}\frac{1}{72\sqrt{70}}\|f^{\left(4\right)}{\|}_{L_{2}}T^{4},\|f^{\prime }-{\left(Q_{T}f\right)}^{\prime }{\|}_{L_{2}}\underset{T\to 0}{∼}\frac{1}{12\sqrt{210}}\|f^{\left(3\right)}{\|}_{L_{2}}T^{3},$$

*for every* $f\in W_{2}^{5}\left(R\right)$ *(cubic B-splines) or* $f\in W_{2}^{11∕2}\left(R\right)$ *(Hermite splines or interlaced derivative sampling). Moreover, in the three cases, the optimal approximation scheme associated to* φ *leads to:*

(69) $$\|f-Q_{T}f{\|}_{L_{2}}\underset{T\to 0}{∼}\sqrt{\frac{10}{3}}\|f-P_{T}f{\|}_{L_{2}},\|f^{\prime }-{\left(Q_{T}f\right)}^{\prime }{\|}_{L_{2}}\underset{T\to 0}{∼}\|f^{\prime }-{\left(P_{T}f\right)}^{\prime }{\|}_{L_{2}},$$

*for every* $f\in W_{2}^{5}\left(R\right)$ *(cubic B-splines) or* $f\in W_{2}^{11∕2}\left(R\right)$ *(Hermite splines and interlaced derivative sampling).*

Proof The three frameworks are readily known to define approximation schemes of order L=4 [13], [37], [41]. All the considered basis functions specify a Riesz basis, are rapidly decaying (Hermite and cubic B-splines are compactly supported while the basis functions for interlaced derivative-sampling, despite being non-compactly supported, are exponentially decaying [41]), and reproduce polynomials up to degree 3. The sampling functions are rapidly decaying generalized functions. Indeed, they are compactly supported for the Hermite and interlaced derivative-sampling schemes. For cubic B-splines, we have seen that $\overset{\sim }{\varphi }=\sum_{k\in Z}{\left(b^{3}\right)}^{-1}\left[k\right]\delta \left(\cdot -k\right)$, where the sequence ${\left(b^{3}\right)}^{-1}$ is known to be exponentially decaying [37], implying the result. In addition, for the Hermite and interlaced derivative sampling schemes (for cubic splines, respectively), (59) is clearly satisfied with p=1 (p=0, respectively). The conditions of Theorem 10 are therefore satisfied for L=4, implying (60).

The value of $E^{\left(2L\right)}\left(0\right)$ is computed by computing the McLaurin expansion of the kernel E.^1^ The analysis of the approximation error on the derivative follows the same principle, the kernel being given for $\left(\varphi^{\prime },{\overset{\sim }{\varphi }}_{\mathrm{int}}\right)$ according to (65), giving (68).

We obtain the asymptotic behavior of the optimal approximation errors $\|f-P_{T}f{\|}_{L_{2}}$ and $\|f^{\prime }-{\left(P_{T}f\right)}^{\prime }{\|}_{L_{2}}$ associated to $\overset{\sim }{\varphi }=\varphi_{d}$ in the same way, leading to (69). □

Our findings call for three comments.

• The three schemes being compared have the same approximation order, the same approximation constant, and the same optimal approximation constant (associated to $\left(\varphi ,\varphi_{d}\right)$) to reconstruct both f and its derivative.
• In all cases, the sampling functions result in a near-optimal asymptotic constant $C_{\varphi ,1}^{\overset{\sim }{\varphi }}$ for the reconstruction of f. The minor discrepancy with respect to optimality is a factor of $\sqrt{10∕3}\approx 1.83$, which is expected from the fact that the sampling functions are not dual functions. It is also remarkable that the reconstruction of the derivative, while not being associated to the dual functions, is associated to an optimal approximation constant $C_{\varphi ,2}^{\overset{\sim }{\varphi }}$.
• Dual functions, although offering the smallest error of approximation, have strong practical disadvantages. First, the $\varphi_{d}$, are non-compactly supported splines. More importantly, they cannot be easily implemented as they do not have a digital filter equivalent (*i.e.*, computation of the $\langle f,\varphi_{d}\rangle$ depend on more than just knowing f and its derivative on a fixed grid, in contrast to usual cubic B-splines and Hermite splines). Finally, dual functions do not possess a closed-form expression in general. For these reasons, the sampling functions $\overset{\sim }{\varphi }$ classically used in the three considered approximation schemes, although non-optimal, are preferable in practice.

In Table 1, we sum up these findings, including the asymptotic constants for cubic B-spline, interlaced derivative sampling, and cubic Hermite splines. We also provide their comparison with optimal constants. Hermite interpolation is thus not a unique way of approximating a function and its first derivative, even if one wishes the error to remain close to optimal. The notable difference lies in the fact that the Hermite scheme provides functions that are simultaneously of finite support, which is not the case for interlaced derivative sampling (see (66) and (67)), and interpolating, which is not the case for cubic B-splines.

## 4 Concluding remarks

Our work focused on the formal investigation of two practical aspects of Hermite splines, namely, their short support and their good approximation properties. We show that Hermite splines are of minimal support among pairs of functions with similar reproduction properties and provide a framework to quantify their power of approximation. These results not only allow us to prove that Hermite splines are asymptomatically identical to cubic B-splines, but also offer a general framework for the quantitative approximation of functions and their derivatives.

**Table 1:** Comparison of approximation methods.

Approximation method	Cubic B-splines	Interlaced derivative sampling	Hermite splines
Digital-filter implementation	✓	✓	✓
Interpolating	✗	✓	✓
Finite support	✓	✗	✓
Closed-form expression	✓	✗	✓

Rate of decay (L)	4	4	4
Asymptotic constant ($C_{\varphi ,1}^{\overset{\sim }{\varphi }}$)	$\frac{1}{72\sqrt{70}}$	$\frac{1}{72\sqrt{70}}$	$\frac{1}{72\sqrt{70}}$
Ratio to optimal ($C_{\varphi ,1}^{\overset{\sim }{\varphi }}$)	$\frac{\sqrt{3}}{\sqrt{10}}$	$\frac{\sqrt{3}}{\sqrt{10}}$	$\frac{\sqrt{3}}{\sqrt{10}}$

Rate of decay (L−1)	3	3	3
Asymptotic constant ($C_{\varphi ,2}^{\overset{\sim }{\varphi }}$)	$\frac{1}{12\sqrt{210}}$	$\frac{1}{12\sqrt{210}}$	$\frac{1}{12\sqrt{210}}$
Ratio to optimal ($C_{\varphi ,2}^{\overset{\sim }{\varphi }}$)	1	1	1

In summary, Hermite splines are found to offer an approximation scheme that (1) has the same approximation power than the notorious cubic B-splines, (2) is interpolating (possibly with the derivative), (3) is based on maximally localized compactly supported basis functions. The resulting cost is the need to use two basis functions instead of a single one, and the need to have access to derivative samples.

## Footnotes

1 We relied on the technical computing software Mathematica 11 for this task.
